# Acetabular Implant Finite Element Simulation with Customised Estimate of Bone Properties

**DOI:** 10.3390/ma16010398

**Published:** 2023-01-01

**Authors:** Dmitriy Soloviev, Leonid Maslov, Mikhail Zhmaylo

**Affiliations:** 1 Institute for Advanced Manufacturing Technologies, Peter the Great St. Petersburg Polytechnic University, 29 Politekhnicheskaya, St. Petersburg 195251, Russia; 2 Department of Theoretical and Applied Mechanics, Ivanovo State Power Engineering University, 34 Rabfakovskaya, Ivanovo 153003, Russia

**Keywords:** computer-aided engineering, hip joint, computer tomography, elastic properties, customised implant, finite element analysis, stress-strain state

## Abstract

The goal of the study is to analyse the strength and stability of a system comprising the pelvis and a customised implant under functional loads using the finite element method. We considered a technique for assessing the elastic properties of bone tissue via computer tomography, constructing finite element models of pelvic bones and a customised endoprosthesis based on the initial geometric models obtained from the National Medical Research Centre for Oncology n.a. N.N. Blokhin (Moscow, Russia). A series of calculations were carried out for the stress-strain state of the biomechanical system during walking, as well as at maximum loads when ascending and descending stairs. The analysis provided conclusions about the strength and stability of the studied device.

## 1. Introduction

**Figure 1:**
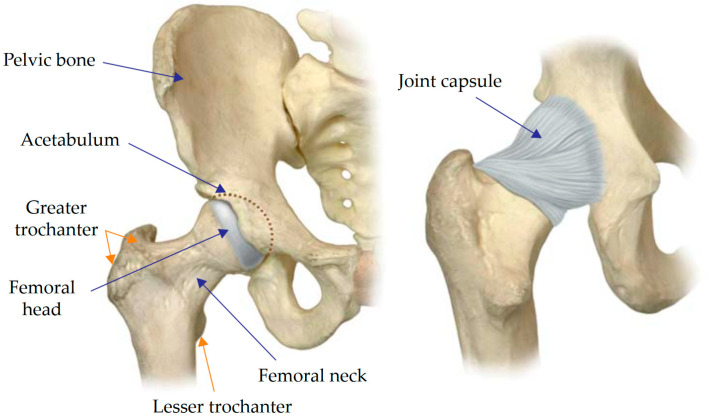
Anatomy of the hip joint [1].

The hip joint is one of the largest joints in the human body, bearing a large share of the loads from walking, running, and weight-carrying (Figure 1) [1]. There are diverse underlying causes behind hip joint disorders, the predominant one being the irreversible breakdown of articular cartilage tissue (osteoarthritis). The number of patients suffering from hip or knee osteoarthritis is steadily increasing.

The demand for customised implants is expected to rise as the population ages and the number of revision (repeated) hip replacement surgeries grows [2]. Degradation of bone and cartilage tissue starts as a result of congenital deformities or injury of the hip joint. Another common cause is a pathology consisting of a tumour developing in bone tissue (sarcoma). Prosthesis implantation helps eliminate or significantly reduce pain and restore motor function in the joint [1].

Customised endoprostheses can provide a solution to accommodate a wide variety of clinical cases, helping to preserve more bone tissue. Treating extensive acetabular defects in revision or oncological hip replacement is very complex: there are numerous options for reconstruction but none has a clear advantage over the others [3,4]. For this reason, a crucial step forward is to evaluate the stability and efficiency of the devices developed, determined by the stress-strain state of the biomechanical system under functional loads.

As rapid progress is made in numerical simulation technologies, new methods for computer-aided design and engineering are developed, capable of predicting the risk of postoperative complications and providing validated decision rules for the choice of endoprosthesis configuration ahead of the surgical procedure. Mechanical stresses exceeding the yield limits may induce the destruction of one or more components, resulting in partial or complete failure of the device. The finite element method (FEM) has proven effective for solving such problems, accounting for heterogeneous structures and complex loading scenarios.

Constructing mathematical models of biomechanical systems can be very time-consuming due to how computer tomography (CT) data are handled, dealing with geometrically complex shapes and the specifics of geometrical representations in the Standard Triangle Language (STL). It is best to avoid oversimplification of the 3D models developed and preserve their anatomical accuracy so as to obtain reliable results.

Novel computing technologies allow for the construction of increasingly large and detailed numerical models yielding increasingly reliable results. For example, numerous studies have emerged over the past two decades on simulations of a healthy and reconstructed pelvis, considering different configurations for the implants and the screws securing them, modelling ligaments, and joints connecting the pelvic bones [5].

The fixation system should be a major focus for customised endoprosthetic devices, since it is the screws that ensure the integrity of the biomechanical system in the postoperative period until osseointegration is complete. For instance, an earlier study [6] considered the effect of the pretension force in the screws anchoring the acetabular component of the hip arthroplasty on the stress state of the pelvic bones and the endoprosthesis component, including the screws themselves. Another study [7] draws a mechanical analogy between dental implants and medical screws, emphasising that keeping the fixation system stable can reduce the risk of fracture in the implant components. The reason for this is that instability in turn leads to fatigue failure in the implant.

The stress-strain state in the healthy and injured pelvis during walking is examined in the publication [8], simulating muscle loads and joint reactions in the AnyBody software and estimating the load transfer across the anterior pelvic ring upon fracture. Experimental studies in the article [9] described a healthy pelvis and two types of prostheses under compressive loads, detecting the asymmetric behaviour of the reconstructed pelvis compared with the healthy one.

Aside from techniques for modelling biomechanical systems and estimating the impact of endoprosthetic designs, another important issue is the reliable measurement of the mechanical properties of bone tissue, which is necessary for constructing highly adequate mathematical models of biological structures. The pelvic bones consist mainly of low-density spongy tissue and a thin cortical layer. Most of the load is transferred through the cortical layer, while the spongy tissue serves as a supporting material, preventing the compact tissue from collapsing. It was established in research [10] that the mechanical properties of bone tissue change drastically with age and the onset of diseases. In addition, the elastic characteristics can vary within the same region of the bone depending on the type of disease.

Since a relatively thin cortical layer carries most of the load, an important strength characteristic to be determined is its thickness. This value varies across anatomical sites in the pelvic bone, also depending on the age of the patient. According to CT data described in article [11], the thicknesses vary in the range from 0.7 to 3.2 mm. The greatest thickness was detected in the superior acetabular rim and the region of the greater sciatic notch extending to the sacroiliac joint. The lowest thicknesses were observed in the sacroiliac joint and the pubic symphysis. According to different sources [12,13,14], the thickness of the cortical layer in the sacrum can vary from 1 to 3 mm.

The degree of elastic anisotropy in the spongy tissue of the pelvic bones was measured in study [15] based on CT and mechanical tests. Young’s modulus and the shear modulus of the bone were found to directly depend on the apparent density. Considering the elastic moduli along the anisotropy axes, the study revealed that the difference between the highest and lowest values was rather small, so bone tissue can be classified as isotropic for simplification.

Some studies [16] compared a healthy pelvis with a reconstructed one, determining the field of elastic moduli based on CT images and finding that some of the screws could be removed from the biomechanical system. A simulation of the sacroiliac joint was carried out in research [17], where the properties of the bone and ligaments were calibrated using experimental data on the applied loads and displacements. Six different configurations for fixing the joint with screws were analysed.

In general, reviewing the data presented in the literature on the mechanical characteristics of pelvic bones, we can observe that there are many factors affecting the elastic properties and their distributions over the bones. The numerical values can vary over a wide range. Therefore, it is essential to develop practical techniques to quickly and reliably assess the physical and mechanical properties of bone based on CT, subsequently using the extracted data to construct patient-specific digital models.

This paper discusses a technique for estimating the distribution and values of elastic parameters of bone tissue obtained from CT data, considering the specifics of finite element models constructed based on initial geometric models generated after preprocessing the CT data. We carried out finite element analysis of the stress-strain state under functional loads and examined the influence of the pretension force in the screws and the absence of screws on the strength and stability of the system consisting of a pelvis and customised implant.

## 2. Materials and Methods

### 2.1. Technique for Evaluating the Elastic Properties of Bone Tissue

Calculating the elastic characteristics of bone tissue is an important aspect of strength evaluation in biomechanical systems. Numerous papers report on data from empirical studies. While these sources provide data on rough estimates for the magnitudes of such quantities as density, ultimate stress, and elastic modulus in the bone, this may prove insufficient. The mechanical properties of bone depend on many factors (age, sex, lifestyle, etc.), and experimental studies tend to yield averaged results. Moreover, the physical and mechanical properties of such anatomical structures as bones are spatially distributed over the entire volume of the bone.

As new technologies for studying biological structures become available, techniques for evaluating the elastic characteristics of bone tissue from CT data are introduced, allowing more accurate mathematical models of the material to be built. Both in-house algorithms [18] and specialised software are used for this purpose (for example, Mimics [16] and Bonemat [19,20]). Grayscale CT images yield more information than just the geometric shape of the anatomical structure. Each pixel in the reconstructed image is assigned a numerical value, expressed as the attenuation coefficient in Hounsfield units (HU), characterising the degree to which an X-ray beam attenuates as it passes through a voxel (unit of volume). A linear transformation is applied to HU values to obtain densities. Thus, this scale describes the approximate density of the substance. The common technique is that the scale of HU values is divided into equal intervals. Most studies assume the bone tissue to be locally isotropic, and each mesh element is assigned an average Young’s modulus obtained using the relationships between density and elastic modulus available in the literature.

The strategy used to average the mechanical properties of bone affects the distribution of the material properties in the model and therefore its accuracy. It was confirmed in article [21] that estimating the mean HU value of an element by simply averaging the HU values in the element nodes or averaging the HU values of CT voxels within each element, as described previously in [22], can yield poor results when the element size is larger or comparable to the voxel size, respectively. An improved averaging algorithm was proposed in research [21] to overcome these difficulties, based on the numerical integration of the HU field over the element volume.

Bonemat software (Bioengineering and Computing Laboratory, Instituto Ortopedico Rizzoli, Bologna, Italy) can import CT images of biological structures and the corresponding finite element (FE) models of these structures, visualise them, establish connections between them and export the FE models, including the meshes with the material properties updated based on the results of CT processing, to some of the most common FE software packages such as ANSYS (Ansys, Inc., Canonsburg, PA, USA) and SIMULIA Abaqus (Dassault Systèmes, Vélizy-Villacoublay, France).

The first step is to import the FE mesh and CT images into Bonemat. A virtual entity representing the input data is created as a result of the import. The user can adjust the position of the visualised slice of the FE model so that it matches the bone in the CT image.

The relationship between HU and density as well as the relationship between density and elastic modulus are specified before the calculations. The former relationship is a linear dependence obtained by scanning the phantom during the calibration of the CT scanner. The latter is a linear or power function that is either constructed independently or, most frequently, selected from the literature. Researchers can also formulate their own relationships between density and Hounsfield units independently, guided by their experience relying on the data from densitometry equipment or measurements for specimens with known densities.

Many examples based on experimental data are given in the literature for the relationships between density and elastic modulus. The form of the relationships may depend on the given anatomical structure (in particular, the relationships are different for the pelvis and femur) and the type of bone tissue (compact or spongy). However, many authors considering the stress-strain state use the same dependence for both types of bone tissue, as done, for example, in publication [18]:

(1) $$\rho_{ash}=0.00063\cdot HU-0.0067\;\left(\frac{g}{cm^{3}}\right),$$

(2) $$E=10500\cdot \rho_{ash}{}^{2.29}\;\left(MPa\right),$$

where $\rho_{ash}$ is the bone ash density, HU denotes Hounsfield units, E is Young’s modulus.

The elastic modulus for spongy bone was assumed to be constant in research [23]. The following relations were used for the cortical layer:

(3) $$\rho_{ash}=0.877\cdot \rho_{CT}+0.079\;\left(\frac{g}{cm^{3}}\right),$$

(4) $$\rho_{app}=\frac{\rho_{ash}}{0.6}\;\left(\frac{g}{cm^{3}}\right),$$

(5) $$E=6850\cdot \rho_{app}{}^{1.49}\;\left(MPa\right),$$

where $\rho_{app}$ is the apparent density of bone.

More complex dependencies are introduced in the study [24]. Three relationships are used depending on the magnitude of the calculated density:

(6) $$\rho_{CT}=10^{-3}\left(0.8\cdot HU\right)\left(\frac{g}{cm^{3}}\right),$$

(7) $$\rho_{ash}=0.877\cdot 1.15\cdot \rho_{CT}+0.08\;\left(\frac{g}{cm^{3}}\right),$$

(8) $$\begin{array}{cc} E_{cort}=10200\cdot \rho_{ash}^{2.01}\;\left(MPa\right),\;\;\; & \rho_{ash}>0.486, \end{array}$$

(9) $$\begin{array}{cc} E_{trab}=2398\;\left(MPa\right),\;\;\; & 0.3<\rho_{ash}\leq 0.486, \end{array}$$

(10) $$\begin{array}{cc} E_{trab}=33900\cdot \rho_{ash}^{2.2}\;\left(MPa\right),\;\;\; & \rho_{ash}\leq 0.3, \end{array}$$

where $\rho_{CT}$ is the calibrated volumetric mineral density of bone or quantitative equivalent CT density, $E_{cort}$ is Young’s modulus for cortical bone, $E_{trab}$ is Young’s modulus for trabecular bone.

A test model was constructed to try out this technique and compare the results with the data available in the literature. It is based on a surface model of the proximal femur and CT images of this anatomical structure from a specific patient, provided by the National Medical Research Centre for Traumatology and Orthopaedics n.a. R.R. Vreden (St. Petersburg, Russia).

In this paper, we adopted the relations proposed by the developers for the test case in Bonemat:

(11) $$\rho_{CT}=0.00079\cdot HU-0.0039\left(\frac{g}{cm^{3}}\right),$$

(12) $$\rho_{ash}=0.877\cdot \rho_{CT}+0.079\;\left(\frac{g}{cm^{3}}\right),$$

(13) $$E=14664\cdot \rho_{ash}^{1.49}\;\left(MPa\right).$$

**Figure 2:**
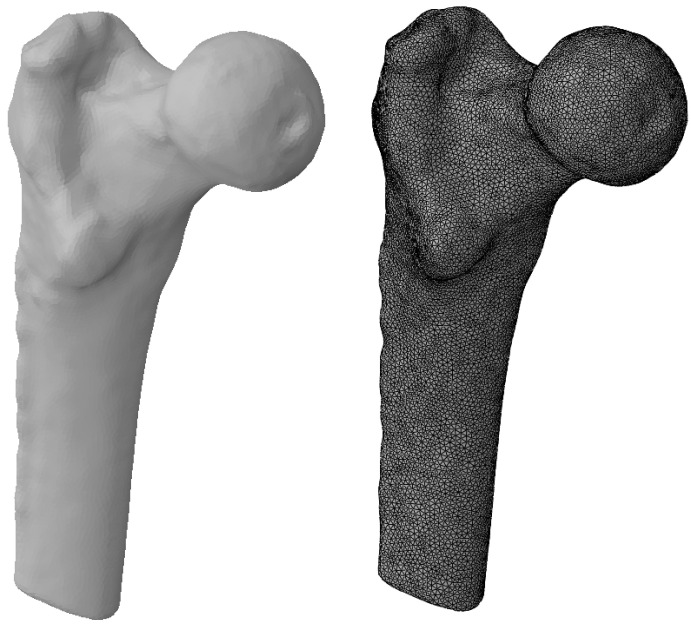
FE model of femur for test problem to evaluate elastic properties.

The bone geometry was carefully processed to reach the closest match between the FE model and the bone tissue in the CT images. The femur was specifically chosen for the test problem because a sufficient number of both experimental and numerical studies evaluating its elastic moduli have been carried out. The FE model is shown in Figure 2.

### 2.2. Description and Characteristics of Customised Endoprosthesis

**Figure 3:**
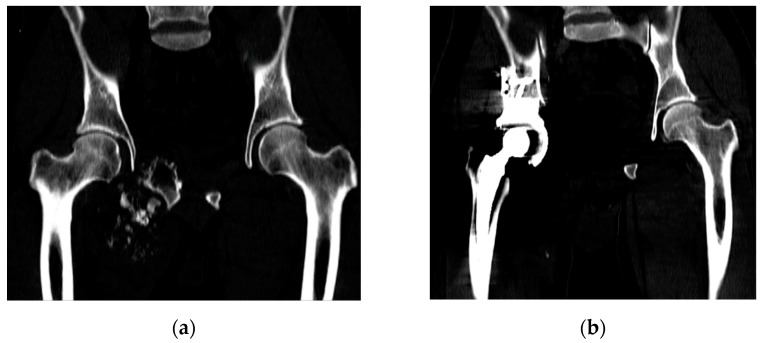
CT images: (**a**) before surgery; (**b**) after surgery.

Our study examines the case of primary total hip replacement (THR). The patient with pelvic osteosarcoma, undergoing treatment at the Blokhin Cancer Research Center, was indicated for the removal of the tumour-invaded pelvic bone structures. A customised endoprosthesis is implanted instead of the right hip joint removed (Figure 3).

**Figure 4:**
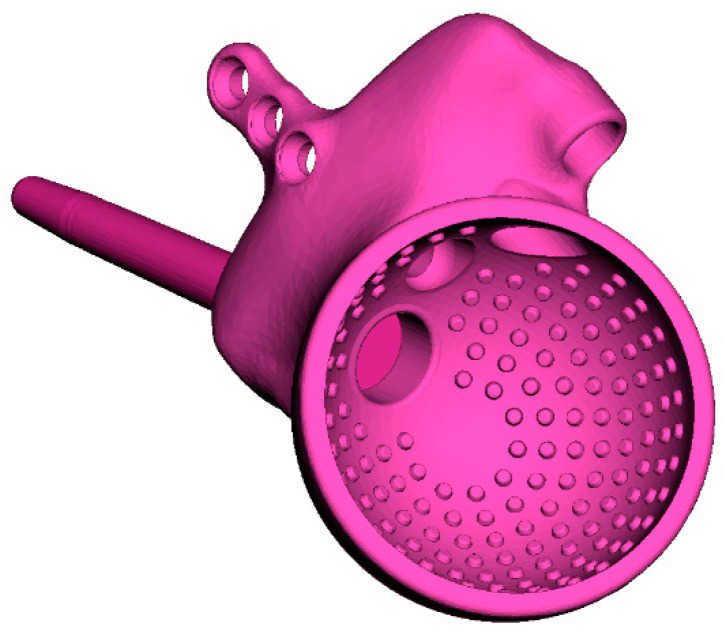
Geometric model of customised endoprosthesis.

The acetabular component in THR is cementless and consists of a cup and a trabecular rod that is inserted into the ilium through a hole in the cup and then sealed with a plug (Figure 4). The endoprosthesis is equipped with an L-shaped flange with holes for titanium screws used to initially fasten the implant to the pelvic bone. The device includes three Ø6.5 mm cancellous screws and three Ø4.5 mm cortical screws.

The inner surfaces of the flange directly contacting the bone are covered with a porous structure to ensure the ingrowth of bone tissue into the endoprosthesis and proper osseointegration. The outer layer of the rod is also made as a porous trabecular-type structure.

Since we did not simulate the liner, head, and plugs in this study, these components are not illustrated in the paper.

The classical equations for a homogeneous linear-elastic solid medium are used as a mathematical model of the implant material. The porous structure was assumed to be a homogeneous solid medium with isotropic effective properties.

The customised endoprosthesis and the screws are made of titanium alloy Ti6Al4V whose main advantages are relatively low density and good corrosion resistance in all conditions. Titanium has excellent biocompatibility in direct contact with tissues or bone. Typical values of mechanical properties are given in Table 1.

The porous structure of the customised endoprosthesis is assumed to be a homogeneous isotropic medium with the effective elastic characteristics of trabecular titanium. This allows the significant reduction of both the computational efforts and the CPU time for solving the problem.

**Figure 5:**
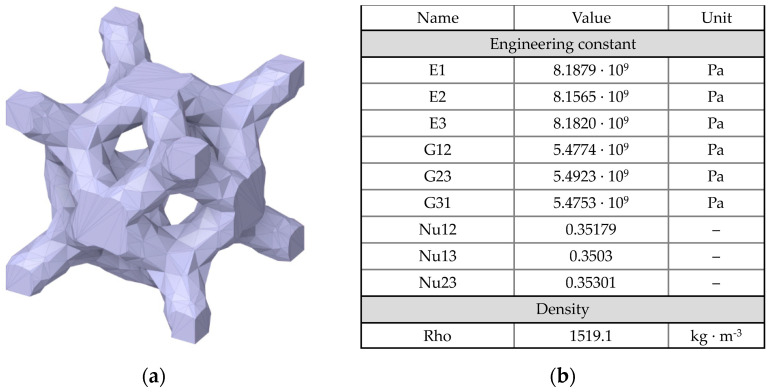
Trabecular titanium: (**a**) unit cell geometry; (**b**) effective characteristics calculated in Material Designer.

The effective mechanical characteristics of the porous structure were determined via the Material Designer module of the ANSYS Workbench 2019 software package (2019). This module allows the importation of the geometry of the unit cell and calculates its effective elastic modulus and Poisson’s ratio. The dimensions of the material’s unit cell were 2.1 × 2.1 × 2.1 mm (Figure 5). The porosity was equal to 65.7%. The values obtained are also given in Table 1.

### 2.3. Finite Element Models of Biomechanical System

Modern digital technologies make it possible to generate fairly accurate 3D models of human internal organs based on data from computer tomography (CT) or magnetic resonance imaging (MRI). Similar functions are provided by software such as Mimics, InVesalius, etc. This technique is very popular at present according to the literature [8,9,16,17,18,25].

**Figure 6:**
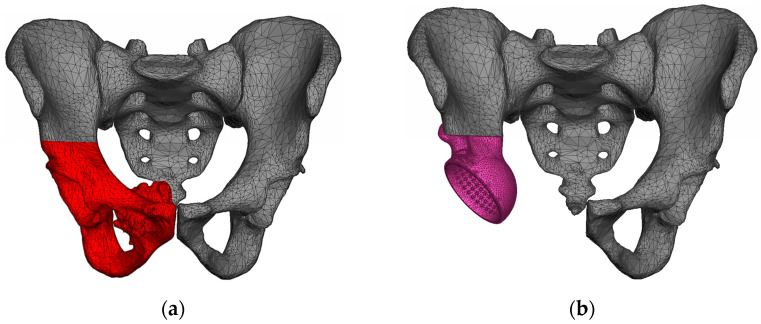
Surface model of pelvis obtained by computer tomography: (**a**) before surgery; (**b**) after surgery.

CT images of the patient’s pelvic region prior to surgery are used for the simulation. Specialised software was used to construct the geometric models of the pelvic bones. The right pelvic bone was divided into two parts corresponding to the planned section of the iliac bone during the surgery. The fragment of the bone marked in red in Figure 6 was completely removed and replaced with the implant. After the reconstructed geometric model was post-processed and positioned, it was converted to STL format.

**Figure 7:**
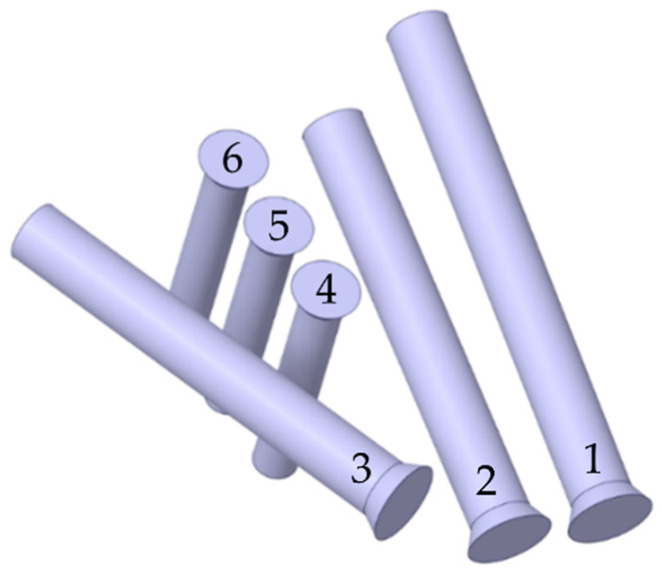
Solid geometry of medical screws and the screw numbering convention.

Next, the geometry files were imported for further processing to the SpaceClaim 2019 software package (Ansys, Inc., Canonsburg, PA, USA, 2019) capable of working with both triangulated surface meshes and solid models. Screws with a simplified geometry were prepared (Figure 7) based on the surface models of the endoprosthesis and the right pelvic bone: each screw consists of a cylindrical body and a truncated cone as the screw head.

A simplified approach to modelling the screws was adopted to keep the resulting complexity of the finite element model within reasonable limits. Of course, describing the shapes of threaded connections directly could improve the model in terms of the amount of valuable results it yields. However, it seems more practical to apply this level of detail to some kind of local model in submodelling analysis. Furthermore, the local results obtained by adopting a very detailed geometry are dependent on the exact angular orientation of each of the screws about their axes, making the results less representative. In view of the above, we decided to use a more simplified shape of the screws in the global analysis carried out.

**Figure 8:**
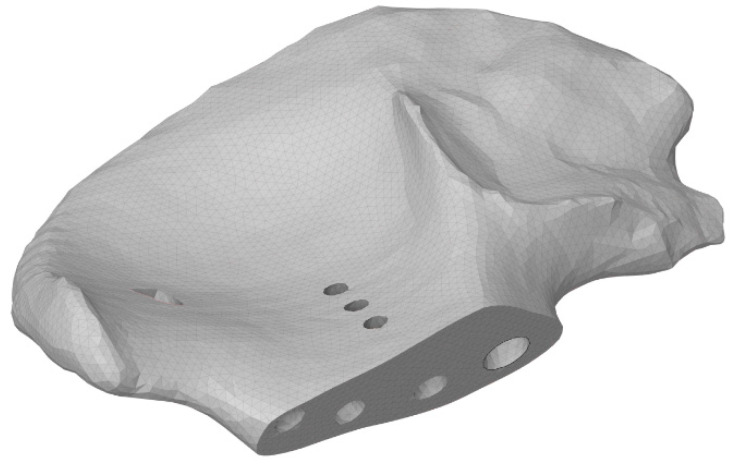
Screw holes in the model of the right pelvic bone.

Before the FE mesh was generated, seven holes were made in the bone tissue (Figure 8) to place the screws and the rod. The surface and volume meshes were prepared with the Altair SimLab 2021 preprocessor (Altair Engineering, Inc., Troy, MI, USA, 2021) capable of handling complex triangulated geometry and performing high-quality remeshing, preserving the shapes of the meshed objects.

**Figure 9:**
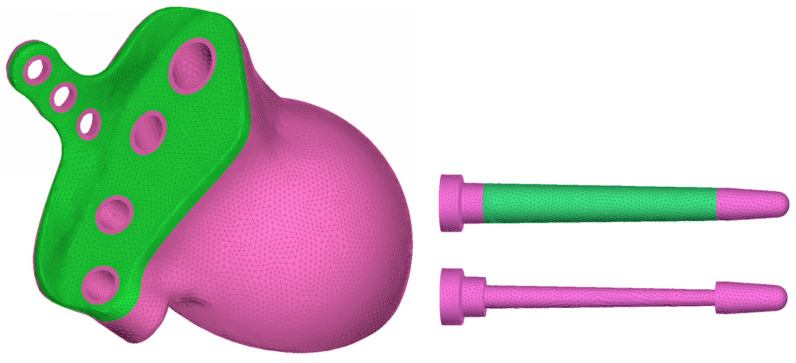
Finite element model of implant.

The computational model of the implant was constructed by the following steps. The surface of the endoprosthesis cup was simplified slightly to reduce the total number of finite elements, as the details of its shape do not significantly affect the stiffness of the model. As mentioned above, the implant is assumed to include a porous structure (trabecular layer), so the endoprosthesis was divided into two volumes that could be assigned different mechanical properties (Figure 9). The thickness of the trabecular layer varies from 2 to 3 mm.

**Figure 10:**
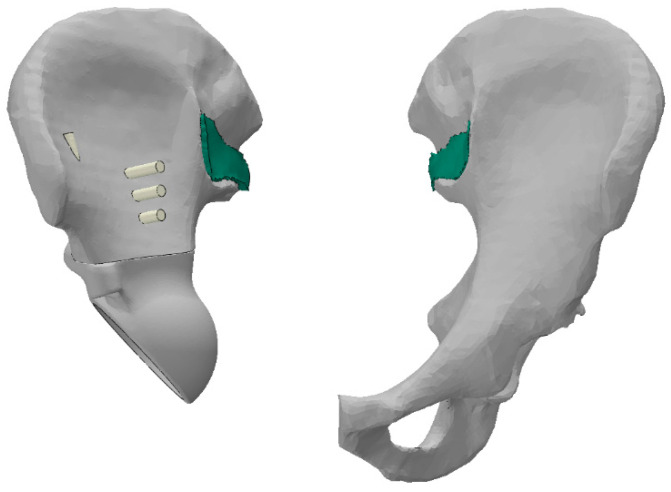
Simulation of sacroiliac joint.

Furthermore, a layer of 2–3 mm thick material is modelled between the sacrum and the pelvic bones, playing the role of a cartilage-covered articular surface in the sacroiliac joint (shown in green in Figure 10). This body is bonded to the sacrum on one side and to the pelvic bone on the other. This approach reduces the stresses from the rigid connections in the bone junctions. Averaged characteristics were selected for the properties of this cartilage based on the literature [5,8,17]: density of 500 kg/m^3^, elastic modulus of 350 MPa, Poisson’s ratio of 0.495.

**Figure 11:**
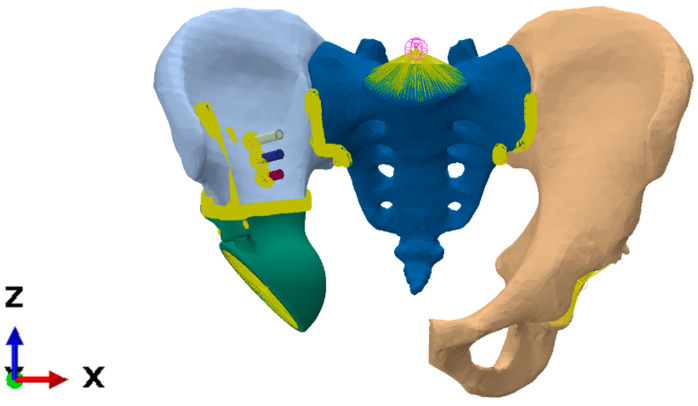
Visualization of interconnected nodes in the finite element model.

The SIMULIA Abaqus CAE 6.14 (2014) software package was used for the assembly of the biomechanical system and finite element analysis. The final model is shown in Figure 11. The nodes of the finite element mesh with the kinematic constraints imposed are marked in yellow.

The model has both bonded connections and frictional contacts. Sets of FE faces of the bodies are selected as contact surfaces. Parts of the screws penetrating the bone are bonded to it, and the screw heads are bonded to the endoprosthesis. The pelvic bones and the sacrum are also connected. Frictional contact acts throughout the entire region of interaction between the endoprosthesis and the bone. The coefficient of friction is 0.6.

### 2.4. Loads and Kinematic Constraints

**Figure 12:**
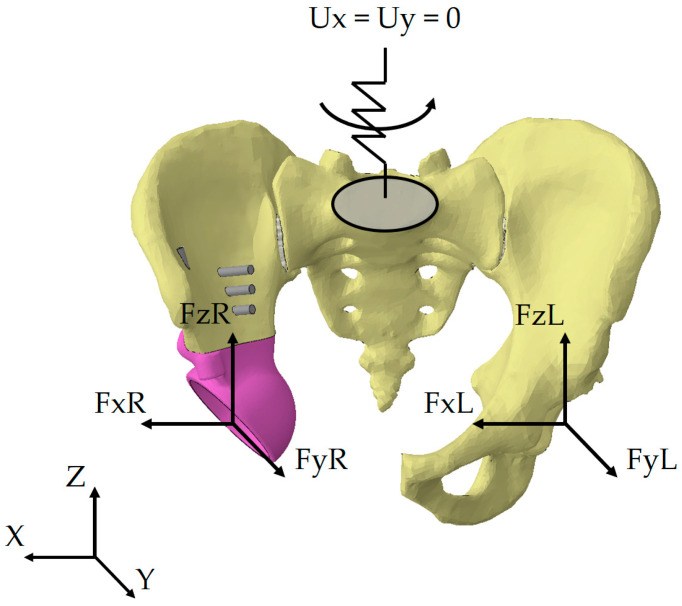
Loading conditions for the pelvis-endoprosthesis system.

The loading scheme is shown in Figure 12, where FxL, FyL, FzL are the reaction forces occurring in the left joint in three projections of the global coordinate system, FxR, FyR, and FzR are the reaction forces occurring in the right joint in three projections of the global coordinate system.

The kinematic boundary conditions for all loading scenarios are represented as a restriction imposed on the displacements of the upper sacral surface. In our earlier works, we used fixation of the top sacrum surface in all three directions [6,25]. Such an approach may negatively affect the stress-strain state of the entire system. Since movements of the lower limbs are always accompanied by periodic vertical displacements of the pelvis and rotations relative to the axes, an additional elastic element was introduced into the mathematical model (Figure 12).

The element introduced has four degrees of freedom: rotations about all three axes and translation along the vertical axis Z. The remaining displacements along the axes X and Y are prohibited. Rigid fixation of the sacrum leads to excessive stress concentrations. Therefore, we introduced additional compliance into the system to avoid this effect.

The stiffnesses of the elastic element were determined from the condition of realistic displacement of the pelvis under the physiological loads arising during normal walking. The following stiffnesses were obtained from the condition that the displacements by the selected degrees of freedom do not exceed 10 mm:

$$\begin{array}{cc} C_{RX}=10^{6}\;\frac{N\cdot mm}{rad};\;\;\; & C_{RY}=10^{7}\;\frac{N\times mm}{rad}; \end{array}$$

$$\begin{array}{cc} C_{RZ}=5\times 10^{5}\;\frac{N\cdot mm}{rad};\;\;\; & C_{UZ}=10^{3}\frac{N}{mm}. \end{array}$$

The external forces acting on the given biomechanical model of the artificial hip joint are divided into two groups: the pretension forces in the screws fastening the implant components and the reactive forces acting from the hip, arising from the person’s motion.

**Figure 13:**
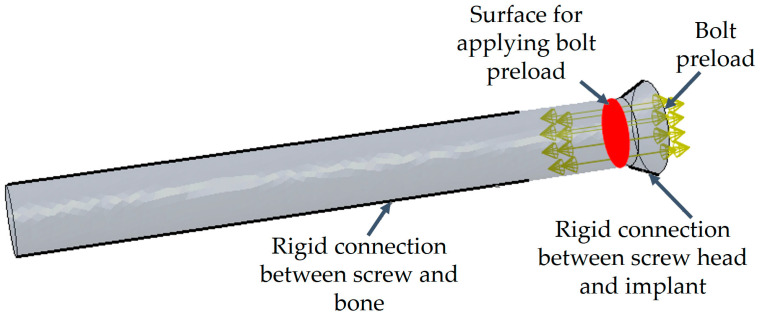
Illustration of force boundary conditions and contact areas for screws.

In the first stage, surgeons pull the implant and bones together with screws. This step is also important in the computational model for obtaining results with higher reliability for the stress-strain state under complex loading conditions. Two values of the pretension force in the screws are considered: 50 N and 500 N. According to the algorithm for calculating the forces in the screw joints, the load is applied to the cross-section of the screw located midway between its head and the region where the implant contacts bone tissue (Figure 13). Both regions of contact are simulated assuming that the surfaces of the bone and segments of the screws are bonded. Sliding contact of rough surfaces is only possible between the surfaces of the implant and the bone in the areas of resection of the pelvic bone and near the rod part of the implant.

The magnitudes of the reaction forces acting from the hip and applied to the geometric centre of the acetabulum in the healthy pelvic area or to the centre of the implant cup in the reconstructed pelvic area are calculated based on the OrthoLoad open database. The HIP98 software [26] (Biomechanics Laboratory, Free University of Berlin, Berlin, Germany, 2011) available at the OrthoLoad portal contains experimental values of reaction forces of the hip joint in the coordinate systems associated with the pelvis or femoral head, occurring in the hip joint during the most common types of everyday activities. The software also visualises the reactions occurring in the joint using graphs and animations for different types of loads: two-legged and one-legged stances, walking at different speeds, standing up and sitting on a chair, ascending and descending the stairs.

The database presents experimental data obtained using specialised endoprostheses in volunteers of different weights and ages. The load magnitudes are obtained using instrumental THR endoprostheses equipped with sensors, as well as external sensors attached to the joints [27]. This equipment allows the recording of the values of the forces occurring in the joint and uploads them to a computer.

**Figure 14:**
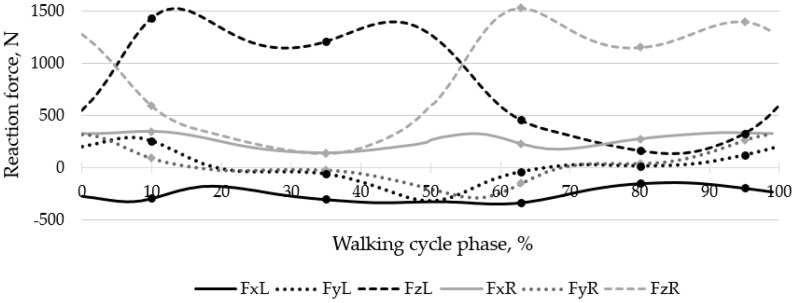
Resultant reaction forces in the hip joint during walking.

Walking simulation is presented as a series of quasi-static analyses with different loads. Five characteristic points were identified in the walking cycle, including the extrema for the right leg, where the endoprosthesis was mounted. Figure 14 shows the resultant reaction forces in the hip joint in the coordinate system of the finite element model, with the selected five points marked. Only the maximum values of loads in the right hip joint were taken from the entire cycle for the cases of ascending and descending stairs (see Table 2).

Loads are given in the database as percentages of the patient’s weight. The specific forces for the given case were calculated for a patient weighing 64 kg. The load directions were adjusted with respect to the local coordinate system of the HIP98 software related to the pelvis and the global coordinate system where the finite element model of the pelvis is considered.

The type of boundary conditions adopted was previously used in other studies [28,29,30]. The approach using diagrams for the reaction forces in the hip joint [26] under complex loading conditions was also successfully tested by other researchers [28,30,31]. This should allow the performance of a qualitative comparison of the results obtained for the stress-strain state with the works where this loading scheme is applied.

## 3. Results

### 3.1. Distribution and Values of Elastic Parameters for Bone Tissue

The distribution of elastic properties of bone tissue was obtained from CT images and specialised Bonemat software using the technique described earlier.

**Figure 15:**
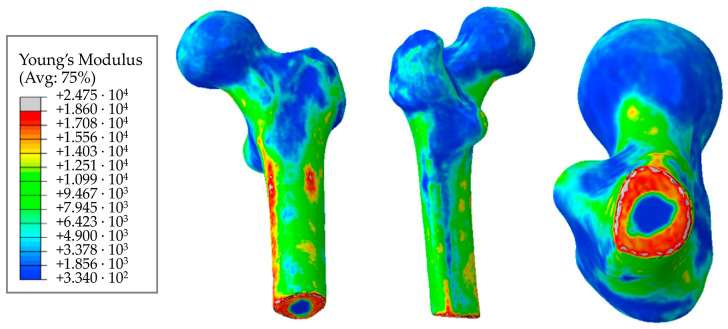
Distribution of elastic moduli in the femur, MPa.

Figure 15 shows the resulting distribution of elastic moduli in the test model of the femur. Visualization was carried out in Abaqus using a script written in Python 2.7.3 (Python Software Foundation, Beaverton, OR, USA, 2010). It is important to mention that the values of the modulus in the femoral neck are predictably higher than in the femoral head.

**Figure 16:**
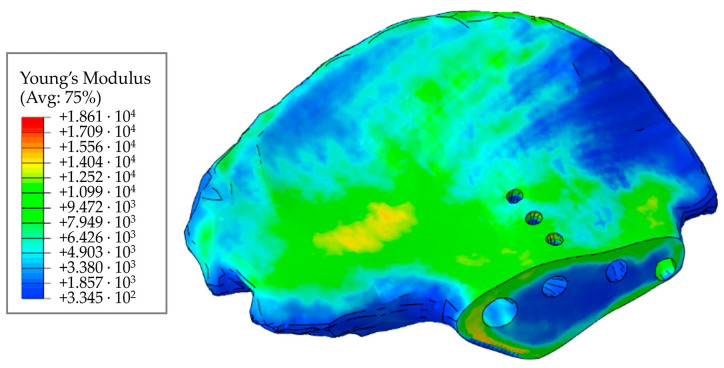
Distribution of elastic moduli in the pelvic bone, MPa.

Figure 16 shows the distribution of elastic moduli for the pelvic bone. We can clearly observe the concentration of increased elastic moduli in the cortical bone layer. Furthermore, comparing Figure 15 and Figure 16, we can conclude that the cortical layer of the femur is stiffer than that of the pelvic bone.

**Figure 17:**
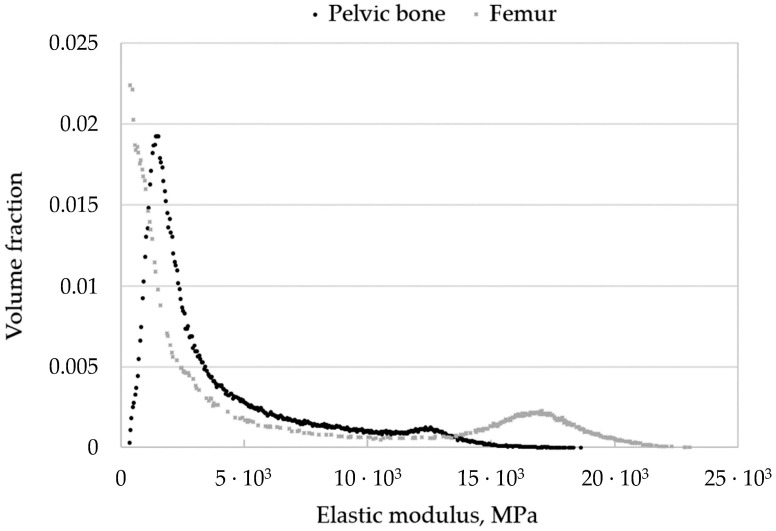
Distribution of elastic moduli over the bone.

Reading and processing the file of the obtained model using Python and Matlab scripts, we divided all the elements comprising the finite element model into a number of sets with similar values of the elastic modulus (Figure 17).

Each set of elements in the finite element model of the pelvis corresponds to a point on the graph (Figure 17). The abscissa of the point is the elastic modulus of the elements in this set, the ordinate is the total volume fraction of elements of the set in the volume of the whole model. It can be seen from the fields that the cortical (densest) layer of bone is more pronounced in the femur than in the pelvic bones, which is to say that the proportion of elements with a higher density is higher.

The final model of the pelvic bone with calculated elastic moduli and densities was transferred to Abaqus for structural analysis.

### 3.2. Stress-Strain Analysis for Screws with the Pretension Force of 500 N

We calculated the stress-strain state at the stage when the screws were tightened, for five extreme values of the reaction forces in the hip joint during walking, as well as for maximum loads when ascending and descending the stairs. The results below are presented separately for screws, endoprosthesis, and bones.

**Figure 18:**
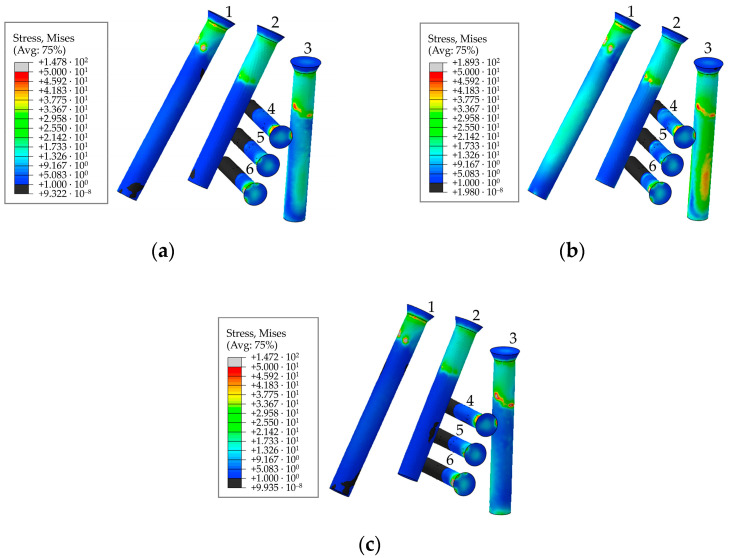
Stress distribution in the screws for a pretension force of 500 N and the screw numbering convention in maximum loading phases corresponding to: (**a**) walking; (**b**) ascending stairs; (**c**) descending stairs, MPa.

The general view of the von Mises stress distribution in the screw system is shown in Figure 18 for a preload of 500 N. The figures show the typical equivalent stress fields occurring at the times (phases) of maximum loading during walking, ascending, and descending stairs. According to Figure 14, the implant replacing the part of the right pelvic bone, including the acetabulum, experienced the greatest loads during walking in the 63% phase of the walking cycle, which corresponds to point 3 in Table 2. Stresses during ascending and descending stairs are calculated and shown only for cases of maximum loads occurring in the right hip joint.

**Figure 19:**
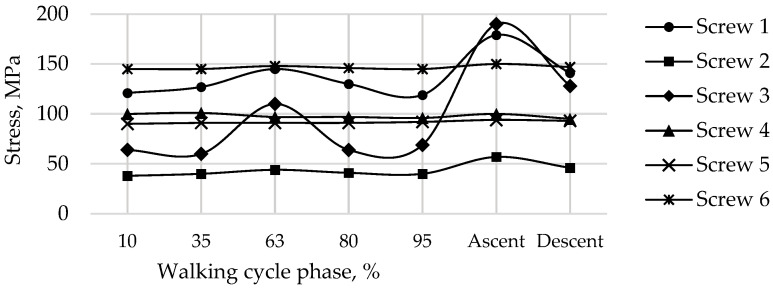
Maximum von Mises stress in each of the screws for a pretension force of 500 N in different phases of the walking cycle.

The graphs show the change in the maximum equivalent von Mises stress in each of the six screws, in the five walking phases, and in the maximum loading phases for ascending and descending stairs (Table 2) at a pretension force of 500 N in the screws are shown in Figure 19.

**Figure 20:**
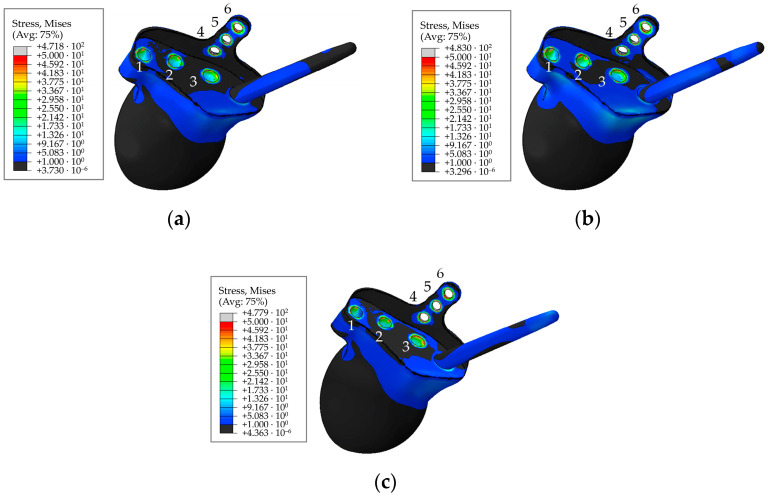
Stress distribution in implant for a pretension force of 500 N and the hole numbering convention: (**a**) In 63% phase; (**b**) for ascending stairs; (**c**) for descending stairs, MPa.

A typical distribution of equivalent von Mises stress in the main component of the hip implant for the pretension force of 500 N in the screws is shown in Figure 20 for the times of maximum loading during the cycle comprising walking, ascending, and descending stairs.

**Figure 21:**
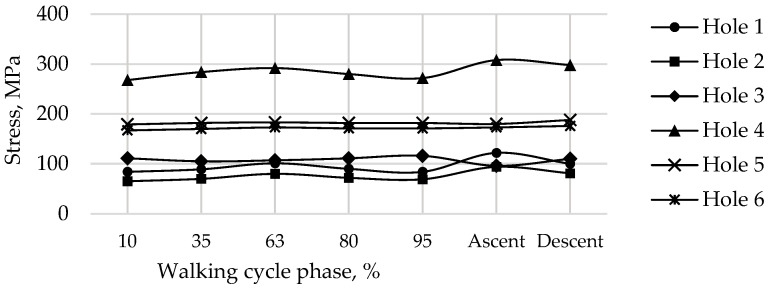
Maximum von Mises stress in the implant at the edges of the holes for a pretension force of 500 N in different phases of the walking cycle.

As seen from the stress distribution in Figure 20, it is particularly important to monitor the stem embedded into the iliac wing and anchoring the implant to the pelvis, and the holes in the flange of the implant for the medical screws. Similar to the screws in Figure 19, Figure 21 shows the equivalent von Mises stress fields with the highest values at the edges of the holes, calculated during the five phases of walking and the maximum loading phases of ascending and descending stairs.

**Figure 22:**
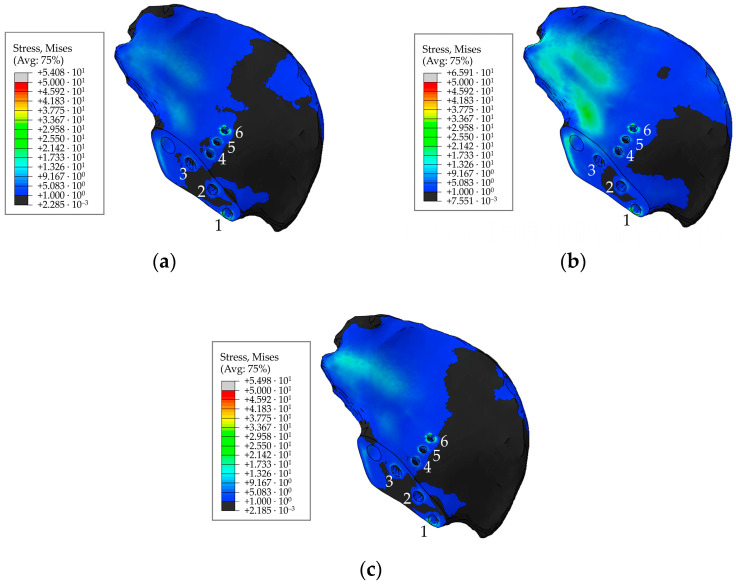
Stress distribution for a pretension force of 500 N and the hole numbering convention in the right pelvic bone: (**a**) in 63% phase; (**b**) for ascending stairs; (**c**) for descending stairs, MPa.

**Figure 23:**
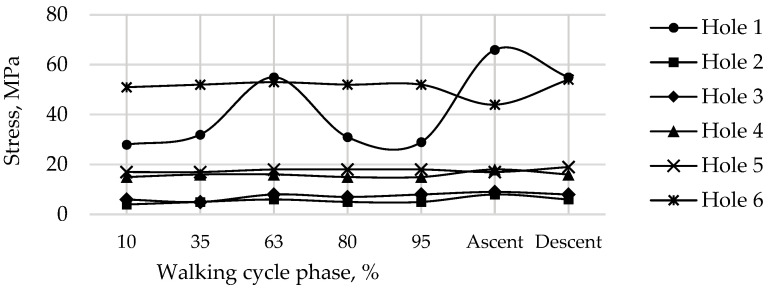
Maximum von Mises stress in the right pelvic bone at the edges of the holes for a pretension force of 500 N in different phases of the walking cycle.

Finally, Figure 22 shows the calculated field of equivalent von Mises stress over the pelvic bone at the times of maximum loads acting on the operated right hip joint during walking, ascending, and descending stairs for a pretension force of 500 N in the screws. Similarly, Figure 23 shows the fields of the equivalent von Mises stress occurring at the edges of the holes drilled in the iliac bone during walking, as well as during the phases of maximum loads during ascending and descending stairs.

Table 3 summarises the maximum stresses detected in the system components in all loading scenarios with a pretension force of 500 N.

### 3.3. Stress-Strain Analysis for Screws with a Pretension Force of 50 N

To assess the influence of the pretension force of the medical screws on the nature of the stress-strain state of the biomechanical system under consideration, calculations were also performed for a preload of 50 N on the screws.

**Figure 24:**
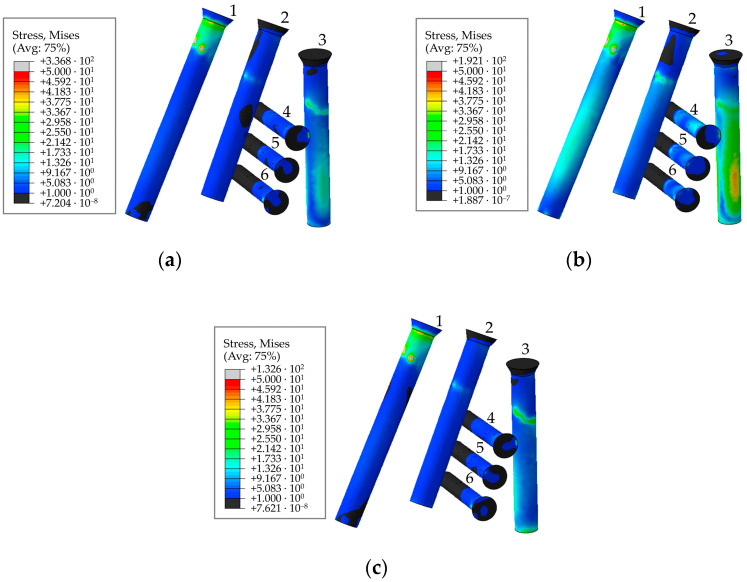
Stress distribution for a pretension force of 50 N and the screw numbering convention in the screws in maximum loading phases corresponding to: (**a**) walking; (**b**) ascending stairs; (**c**) descending stairs, MPa.

**Figure 25:**
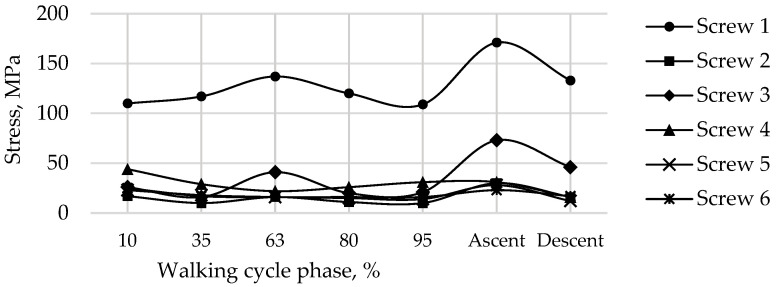
Maximum von Mises stress in each of the screws for pretension force of 50 N in different phases of the walking cycle.

Similarly to the previous case, Figure 24 and Figure 25 show the fields of equivalent von Mises stress in the medical screws and at the edges of the holes in the implant, calculated during the characteristic phases of the walking cycle (Figure 14) and times of maximum loads in the right hip joint (Table 2) during ascending and descending stairs.

**Figure 26:**
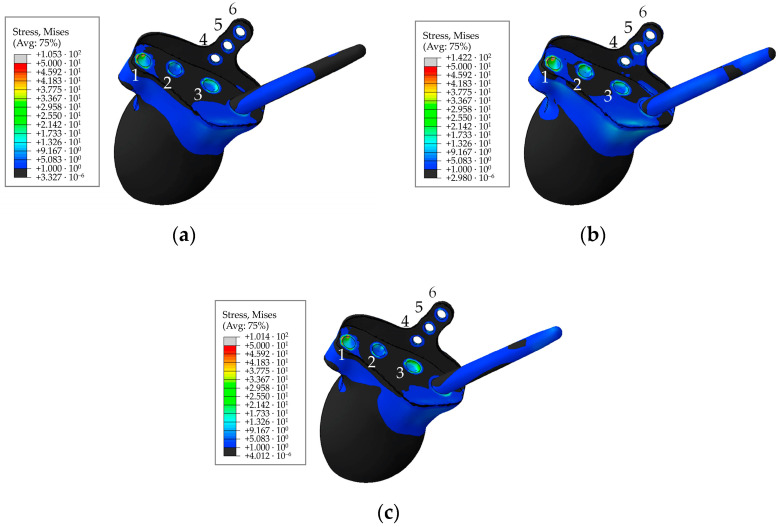
Stress distribution for a pretension force of 50 N and the hole numbering convention in implant: (**a**) in 63% phase; (**b**) for ascending stairs; (**c**) for descending stairs, MPa.

**Figure 27:**
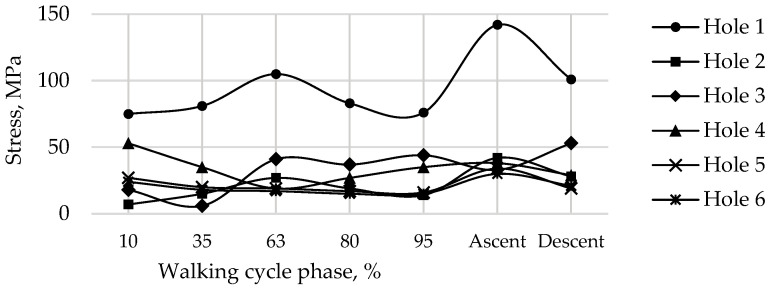
Maximum von Mises stress in the implant at the edges of the holes for pretension force of 50 N in different phases of the walking cycle.

Figure 26 and Figure 27 show the distribution of equivalent von Mises stress in the endoprosthesis components (screws and implant) for the phases of maximum loading of the right hip joint in the cycles of walking, ascending, and descending stairs.

**Figure 28:**
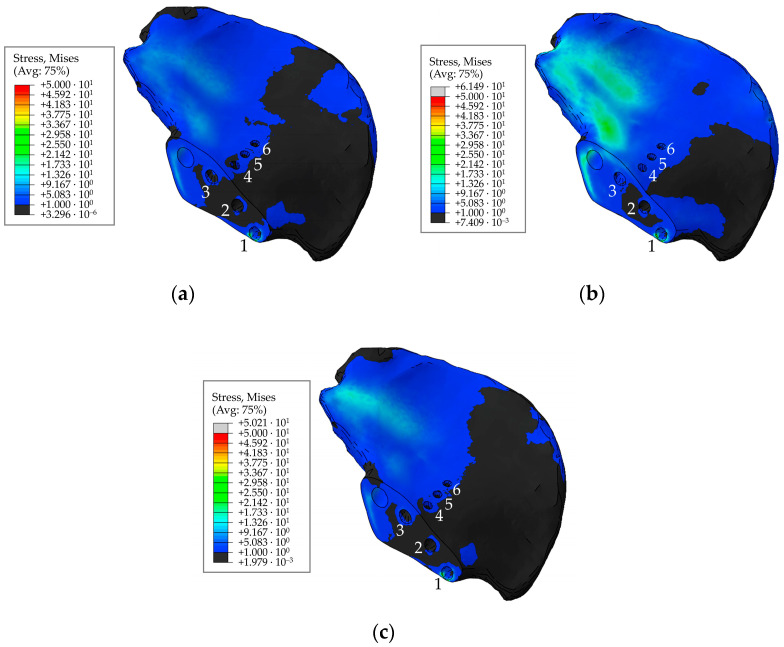
Stress distribution for a pretension force of 50 N and the hole numbering convention in the right pelvic bone: (**a**) in 63% phase; (**b**) for ascending stairs; (**c**) for descending stairs, MPa.

**Figure 29:**
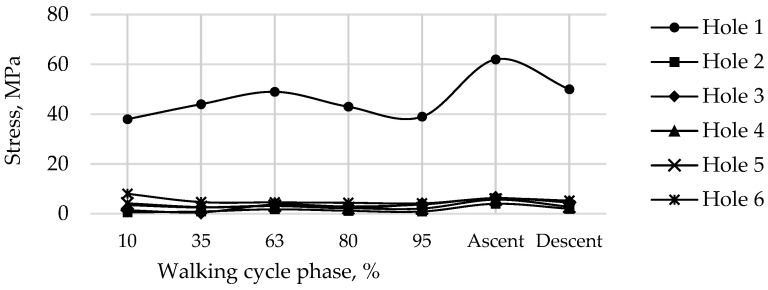
Maximum von Mises stress in the right pelvic bone at the edges of the holes for pretension force of 50 N in different phases of the walking cycle.

Finally, the distribution of equivalent von Mises stress in the pelvic bone material at a pretension force of 50 N is shown in Figure 28 at the times of maximum loads acting on the operated right hip joint during walking, ascending and descending stairs. Figure 29 shows the fields of equivalent von Mises stress in the bone tissue in the regions of the medical screw holes.

The overall results for the pretension force of 50 N are summarised in Table 4.

### 3.4. Contact Characteristics Analysis

**Figure 30:**
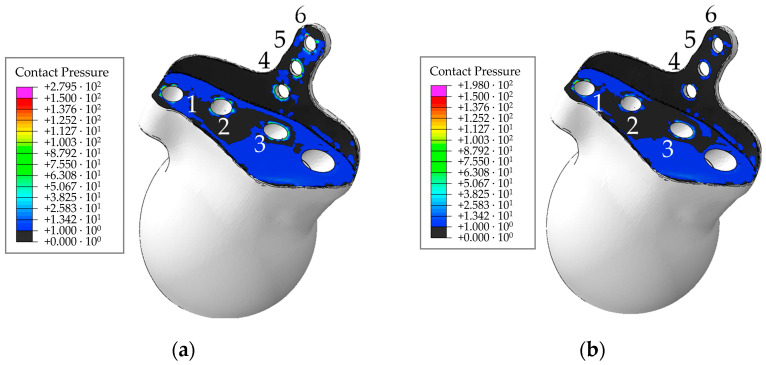
Contact pressure and the hole numbering convention for the walking cycle: (**a**) for a pretension force of 500 N; (**b**) for a pretension force of 50 N, MPa.

Let us analyse the area of contact between the endoprosthesis and the bone. Figure 30 shows the field of maximum contact pressure over all loading steps for pretension forces of 500 N and 50 N.

**Figure 31:**
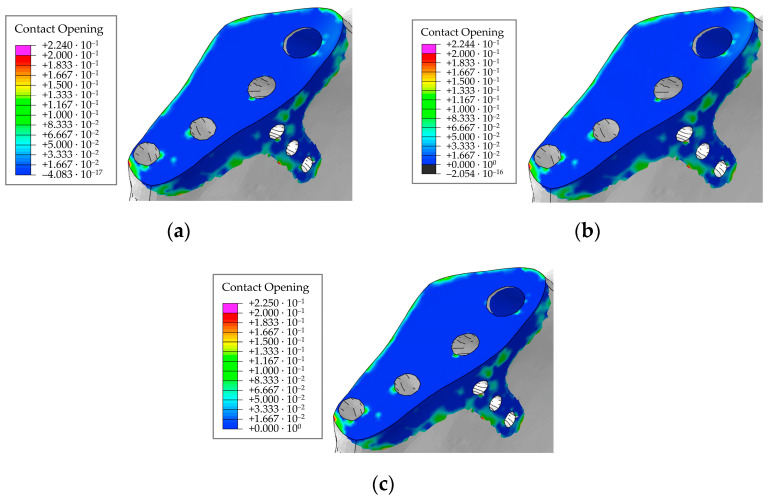
Maximum clearance between the contact surfaces over all loading steps: (**a**) for pretension force of 500 N; (**b**) for pretension force of 50 N; (**c**) initial state, MPa.

Next, we also evaluated the field of maximum contact opening between the contact surfaces (COPEN parameter in Abaqus) over all loading steps. The calculation results are shown in Figure 31 for the times corresponding to the maximum loads in the right hip joint during walking, ascending, and descending stairs at two values of screw pretension forces of 500 N and 50 N, and for the initial condition of contact between the implant and the bone without any screw tightening.

## 4. Discussion

Reliable quantitative assessment of mechanical properties of biological tissues is critically important to be able to adequately calculate the stress-strain state of complex biomechanical systems. This is a matter of general consensus in discussions about the application of the finite element method to solving the problems of tissue biomechanics.

A known issue is that while artificial materials are made of well-examined components following exact technological procedures, it is very difficult to determine the exact values of elastic moduli for living tissues. This is especially important for bone tissues, both cortical and spongy, due to their complex internal structure, which is variable over the volume of the bone. Another complication is that the mechanical properties of living tissues can be drastically different in different people, depending on a person’s lifestyle changing over time.

Thus, the values of elastic moduli obtained by the common experimental methods in rather large samples of nonliving tissues cut from random skeletal specimens, which are widely used in publications, cannot be considered sufficiently reliable for developing digital twins and performing virtual testing of biomechanical systems. High-resolution computer tomography can be combined with the mathematical processing of images to tackle many of the existing challenges, so this approach should be adopted in research to accumulate representative data.

The results we obtained in this study for the elastic moduli of bone tissues have a clear physical meaning and are in good agreement with the known quantitative estimates. In particular, the distributions of elastic moduli in Figure 15 and Figure 16 confirm significantly higher values of elastic properties of the cortical tissue making up the long tubular bones of the human skeleton, as compared to the cortical layer of the pelvic bones. The reason for this may lie in the peculiarities of accommodation and transfer of mechanical loads by these bone structures. The obtained distributions of elastic moduli for both the femoral (Figure 15) and the iliac (Figure 16) bones correspond to the known patterns of the force lines of the principal stress.

The computer technologies used in this work allowed to geometrically separate the dense cortical tissue with high elastic moduli from spongy tissue with low densities and low elastic moduli. An adequate uniform distribution of elastic moduli over the bone volume was obtained, excluding jumps in elastic moduli absent in living tissue.

The elastic modulus given in the literature for the human femur varies on average from 1 GPa to 18–20 GPa [20,32,33]. The elastic moduli in article [34], obtained similarly, range from 500 MPa to 17,000 MPa. The moduli given in the study [20] range from 50 MPa to 20,000 MPa. Furthermore, a value of 22.5 GPa was obtained in research [35] for the cortical layer of the femur.

Comparing these values with those obtained in our study by Equations (11)–(13), we can assume that the calculated maximum values (21–23 GPa) are somewhat overestimated. However, the volume fraction of such elements in the model is small (Figure 17). Moreover, it can be seen from the obtained fields that the cortical (densest) layer of bone is more pronounced in the femur than in the pelvic bones, which is to say that the proportion of elements with a higher density is higher.

The elastic modulus for the pelvic bone calculated in this study varies from 330 MPa to 18,600 MPa. In general, the results obtained are consistent with the experimental data [36].

Therefore, this technology can yield a more accurate model of bone tissue material matching a particular patient and accounting for individual characteristics.

For a more convenient assessment and comparison of the obtained results of the structural analyses, a summary of the maximum equivalent stress occurring in the implant parts and bone tissue is presented in Table 5 for the two considered values for the screw pretension loads of 50 N and 500 N.

The most loaded phase for the implant and screws during walking is the 63% phase, since the maximum value of the reaction force for the right hip joint occurs in this period. The ascent was the most loaded among all computational cases, the most loaded screw was Screw 3. The curves of maximum stress for Screws 3 and 4 are shown in Figure 19. Evidently, Screw 4 takes the most load at the tightening stage already, and the stresses remain mostly unchanged after that, which seems to be relatively predictable. Similar results were obtained for Screws 5 and 6. Hypothetically, one of these screws can be removed provided that the stability of the system is preserved.

Stresses in the rod, as well as at the edges of the holes are shown in Figure 20 and Figure 21. The maximum stresses in the endoprosthesis are significantly lower than the critical stress (950 MPa for titanium [37]) and do not exceed 500 MPa. The most loaded sections are the edges of the screw holes. The rod turned out to be lightly loaded. The maximum stresses in it are not higher than 40 MPa during the walking cycle, and do not exceed 15 MPa in the trabecular structure. Stresses during ascent reach 85 MPa, and 30 MPa in the trabecular structure.

The stresses in the bone are also concentrated near the holes and do not exceed 70 MPa (Figure 22 and Figure 23). The highest values for the walking cycle are observed in the area of contact between the bone and the implant around Hole 1 and Hole 6. Furthermore, the region near Hole 1 appears dangerous due to the small thickness of the remaining bone, but the maximum stresses in it do not exceed the tensile strength of the cortical bone (100–150 MPa [36]).

The case of ascent also remains the most loaded case. The case of descent is similar to the 63% phase of the walking cycle in terms of stress values. The variation in the stress state in the area of contact between the bone and the endoprosthesis depending on the given load is less pronounced than in the remaining regions of the model.

The distribution of displacements and stresses in the system does not change if the pretension force of the screws is reduced to 50 N (Figure 24, Figure 25, Figure 26 and Figure 27). Variations in the stress-strain state are observed in the area near the screws, the variations in other regions of the model are insignificant. The stresses in the screws and implant decrease near the screw holes. The stresses in the endoprosthesis remain mostly unchanged near Hole 1 and in the rod. Stresses at the edge of Hole 3 decrease by more than two times.

No stress concentrations arise around the holes in the bone under functional loads (Figure 28 and Figure 29). The stresses are considerably decreased at the edge of Hole 1 only at the stage of tightening the screws. This area may still be dangerous. The stresses are significantly reduced near other holes, becoming negligible in the general stress state. The question is therefore whether the device will remain stable during walking if the screws are loosely tightened. If the area of contact between the bone and the trabecular structure of the endoprosthesis is constantly loosened, this may have a negative effect on the osseointegration of the implant.

We should mention that the stresses in the hole drilled in the bone for the implant rod do not depend on the pretension force in the screws and vary from 5 to 12 MPa during the walking cycle. The maximum stress values for the cases of ascent and descent are 31 and 10 MPa, respectively.

Analysis of the contact behaviour between the surfaces of the implant and the bone showed that a significant decrease in pressure is observed for the force of 50 N, the pressures are less than 1 MPa for the most part of the contact area (Figure 30). Local increases in pressure are observed near the screws. The pressure remains the same and is within 20 MPa in the rest of the contact area.

The maximum opening is 0.22 mm regardless of the pretension force, not changing when the patient moves (Figure 31). Thus, the contact area remains stable in the presence of screws under functional loads during walking. Since the geometry of the model is not “perfect”, the contacting surfaces do not have a tight fit to each other over the entire contact area. Therefore, initially, there are some regions with open contact in the bone/implant contact pair (Figure 31). The gap in the contact varies insignificantly over all loading steps compared with the initial conditions.

Contact analysis indicates that there should be no significant problems with the stability of the biomechanical system because the design of the endoprosthesis is quite efficient (in particular, due to the rod) and the implant is pressed tightly to the bone by the reaction forces in the hip joint. The risk of osseointegration failure increases with decreasing pretension force in the screws [38]. Indeed, as no stresses leading to destruction are observed in the case of a pretension force of 500 N, it is preferable to select this value of the pretension force in the screws.

It is relatively difficult to directly compare the obtained results with the publications of other authors because the endoprosthetic device is personalised. Nevertheless, the qualitative picture of stress distribution in the pelvis/endoprosthesis system and the average level of stress state both in the implant material and in the bone tissue are in general agreement with the numerical values given in similar publications [5,6,8,9,16,24,25,28,29]. For instance, it is established in work [8] that the equivalent von Mises stress of about 20–40 MPa occurs during walking in the intact pelvic bone, with the highest stresses occurring near the sacrum. Our results generally confirm both the level of stresses in the pelvic bones and the rise in values in the sacral region. However, the holes in the bone tissue exhibit a pronounced stress concentration around the edges, reaching up to 100 MPa when walking and 150 MPa when ascending stairs.

As for the stress state in the endoprosthesis components, it seems reasonable to compare the stresses in the screws with the data given in study [6] because the problem was formulated similarly in that study and the endoprosthesis had a similar design. The stresses in medical screws during walking at a pretension force of 500 N given in [6] vary from 50 MPa to 150 MPa depending on their diameter but in some cases reach almost 200 MPa. The stresses found in the screws in our study are generally much lower. In our opinion, this is due to the modified design of the personalised endoprosthesis: specifically, a rod anchoring the implant in the wing of the iliac bone was added. This means that unlike the endoprosthesis in publication [6], which was attached with screws only, the fixation rod in the new device takes on some fraction of the load, providing a more uniform distribution of stresses over the endoprosthesis components and ultimately serving to reduce the stresses.

Other types of endoprostheses (see, for example, in articles [28,29]) induce a stress state in the implant that is close to the obtained results. For example, a rather complex customised hip endoprosthesis is constructed and analysed in study [29]: the stresses at maximum loads simulating human walking are about 130–170 MPa in different components of the implant. Notably, the level of stress in the structure considered in our study is much lower, except for the areas of concentration near the screws, which should be taken into account in design and operation of endoprostheses mounted with screws.

The effects of the pretension force in medical screws are studied in research [28], which calculates that the peak von Mises stress in a 3D-printed Ti6Al4V augment ranges from 10 MPa at a 500 N pretension force to 61 MPa at a 3000 N pretension force, and the stresses in the screws range from 12 MPa to 76 MPa, respectively. However, no studies using human motion are available.

In conclusion, we should mention that the approach developed based on the known measurements of reaction forces in the hip joint [26,27] has a simpler and better validated formulation compared to research [8]. This way, finite element analysis of stresses can be extended quite easily to different cases of the patient’s motions and, accordingly, yield better estimates for the strength of the biomechanical structure. This presents a clear advantage of the results obtained by the approach adopted in comparison with the results obtained by calculations of the stress-strain state in the hip joint under the weight of a person standing statically on two legs, as done, for example, in [9,16,25]. At the same time, it is found in research [16] that the forces acting on the hip joint during walking and other types of human motion, especially running and jumping, can increase by several times, which confirms the significance of the results we have obtained in this study.

## 5. Conclusions

The paper presents a review of the literature dedicated to the simulation of the human pelvis under physiological loading. Processing the surface models for the components of the system comprising the pelvis and the customised implant, we built models of the screws and generated finite element meshes. We have considered different aspects related to constructing the models and the requirements imposed on them.

A specialised technique was devised for estimating the elastic moduli distribution of bone tissue, detected by computer tomography. Reviewing the literature data, we have confirmed that the properties obtained for the bone can be deemed satisfactory. We believe that the constructed model of bone tissue material is more realistic compared with earlier studies, as it can account for the individual characteristics of a specific patient.

A finite element model of the system comprising the pelvic bones and the customised endoprosthesis was generated in the Abaqus software package. A series of calculations were carried out for the stress-strain state of the biomechanical system during walking, as well as at maximum loads when ascending and descending stairs. The endoprosthesis and the screws have almost twice the safety margin compared to the corresponding critical stress. The edge of Hole 1 in the right pelvic bone appears to be the most dangerous region because the remaining bone has a small thickness: the stresses here do not exceed the yield limits but are close to them. We can thus assume that the implant design is effective in terms of structural strength and stability within the framework of the problem statement considered.

## Figures and Tables

**Table 1:** Physical and mechanical properties of implant material.

Material	*ρ*, kg/m^3^	Elasticity	
		*E*, GPa	*Ν*
Titanium alloy Ti6Al4V	4430	113.8	0.342
Trabecular titanium (65.7% porosity)	1520	818	0.35

**Table 2:** Calculated reaction forces in the hip joint, in three projections for both legs.

Points	Load Magnitudes in Projections on the Axis, N					
	Right Leg			Left Leg		
	FX	FY	FZ	FX	FY	FZ
Walking, point 1	345	91	593	−292	255	1434
Walking, point 2	139	−22	134	−302	−63	1211
Walking, point 3	225	−151	1530	−332	−40	456
Walking, point 4	273	40	1152	−150	16	163
Walking, point 5	330	262	1397	−194	121	325
Ascending stairs	125	−593	1635	−324	−17	604
Descending stairs	184	−5	2001	−352	−76	577

**Table 3:** Maximum von Mises stress in the system components with pretension force of 500 N.

	Maximum von Mises Stress in Contact Pair, MPa					
Position	Hole					
	1	2	3	4	5	6
Holes in bone tissue	66	84	9	18	19	54
Holes in implant	122	94	116	308	188	176
Screws	141	46	128	95	93	147

**Table 4:** Maximum von Mises stress in the system components with pretension force of 50 N.

	Maximum von Mises Stress in Contact Pair, MPa					
Position	Hole					
	1	2	3	4	5	6
Holes in bone tissue	62	4	6	6	6	8
Holes in implant	142	42	53	53	34	30
Screws	171	30	73	44	28	25

**Table 5:** Summary of the maximum von Mises stress occurring in the endoprosthesis components and bone tissue for screw pretension loads equal to 50 N and 500 N.

Components		Maximum von Mises Stress and Their Location				
	Pretension Force of 50 N			Pretension Force of 500 N		
	Walking Cycle	Ascending Stairs	Descending Stairs	Walking Cycle	Ascending Stairs	Descending Stairs
Screws	137 MPa, screw 1	171 MPa, screw 1	133 MPa, screw 1	148 MPa, screw 6	190 MPa, screw 3	147 MPa, screw 6
Implant	105 MPa, hole 1	142 MPa, hole 1	101 MPa, hole 1	292 MPa, hole 4	308 MPa, hole 4	298 MPa, hole 4
Right pelvic bone	49 MPa, hole 1	62 MPa, hole 1	50 MPa, hole 1	55 MPa, holes 1 and 6	66 MPa, hole 1	55 MPa, holes 1 and 6

## Data Availability

The data presented in this study are available on request from the corresponding author.
